# The prevalence of type 2 diabetes in people with psychiatric disorders: an umbrella review protocol

**DOI:** 10.1186/s13643-020-01341-6

**Published:** 2020-05-04

**Authors:** N. Lindekilde, S. Scheuer, F. Rutters, J. E. Henriksen, K. H. Rubin, M. Lasgaard, G. S. Andersen, F. Pouwer

**Affiliations:** 1 Department of Psychology, University of Southern Denmark, Campusvej 55, 5230 Odense M, Denmark; 2 Clinical Epidemiology, STENO Diabetes Center Copenhagen, Gentofte, Denmark; 3 Department of Epidemiology and Biostatistics, Amsterdam Public Health Institute, Amsterdam UMC, location VUMC, Amsterdam, The Netherlands; 4 STENO Diabetes Center Odense, Odense University Hospital, Odense, Denmark; 5 OPEN - Open Patient data Explorative Network, Department of Clinical Research, University of Southern Denmark and Odense University Hospital, Odense, Denmark; 6 DEFACTUM - Public Health & Health Services Research, Central Denmark Region, Aarhus, Denmark; 7 School of Psychology, Deakin University, Geelong, Australia

**Keywords:** Type 2 diabetes, Psychiatric disorder, Prevalence, Umbrella review, Systematic review

## Abstract

**Background:**

Many epidemiological studies have investigated the prevalence of type 2 diabetes in individuals with a psychiatric disorder. In an umbrella review, we aim to systematically summarize existing systematic reviews examining the prevalence of type 2 diabetes in people with a psychiatric disorder. When information is available in the identified systematic reviews, comparisons with control groups without a psychiatric disorder will be made. Furthermore, we aim to assess the quality of the included systematic reviews.

**Methods:**

The umbrella review will be based on a comprehensive systematic search of systematic reviews of observational (cross-sectional or longitudinal) studies investigating the prevalence of type 2 diabetes in people with a psychiatric disorder. Four electronic databases (Embase, PsycINFO, PubMed, and the Cochrane Database of Systematic Reviews) will be searched. Retrieved papers will be screened for eligibility by two independent reviewers. Furthermore, the reference lists of all included publications will be screened. Data will be extracted by using an a priori developed data extraction form and two independent reviewers will assess the risk of bias in the included systematic reviews using with the Risk of Bias in Systematic Reviews (ROBIS) tool. A narrative data-synthesis and a subsequent meta-analysis based on the primary studies will be made.

**Discussion:**

For each psychiatric disorder, the data regarding the prevalence of type 2 diabetes will be summarized and discussed. When possible, comparisons with control groups will be reported and discussed. Finally, future implications and recommendations for clinical care will be presented.

**Systematic review registration:**

This protocol was submitted for registration with the International Prospective Register of Systematic Reviews (PROSPERO) on December 9, 2019 (registration number: pending).

## Background

Psychiatric disorders are common conditions. According to a systematic review from 2014, almost 1 in 5 adults worldwide meet the criteria for a psychiatric disorder during a 12-month period [1]. The life-time prevalence of psychiatric disorders was 29% (26–33%) [1]. Different psychiatric disorders do not only seriously impact the quality of life of those affected and their family members, the disorders may also have a negative impact on other health outcomes. For example, a systematic review of longitudinal studies showed that mortality rates were significantly higher in people with psychiatric disorders than in those without such disorders (pooled relative risk = 2.22; 95% CI, 2.12–2.33; *n* = 133 studies) [2]. Furthermore, the median years of potential life lost was 10 years (*n* = 24 studies) [2].

It is important to better understand how different psychiatric disorders can impact long-term health outcomes. One adverse outcome is diabetes, which is a chronic metabolic condition that affected approximately 463 million people worldwide in 2019 [3]. Ninety percent of all people with diabetes are diagnosed with type 2 diabetes [4]. Many studies have shown a link between psychiatric disorders and type 2 diabetes [5–7]. In the last decades, complex associations between psychiatric disorders and type 2 diabetes have been theorized and investigated. For example, in 2014, de Jonge et al. determined the association in a large cross-national study that used diagnostic psychiatric interviews and data on self-reported diabetes. In this study, after adjusting for co-morbid psychiatric disorders, particularly depression, intermittent explosive disorder, binge eating disorder, and bulimia nervosa were associated with diabetes [5]. For several psychiatric disorders, it has been postulated that they have a bidirectional association with type 2 diabetes [6, 7]. Already 10 years ago, De Hert et al. described diabetes and cardiovascular diseases as significant issues in patients with severe mental illness [8]. However, the care for diabetes in people with psychiatric disorders remains far from optimal [9]. A recent Danish study indicated lower quality of diabetes care in people with diabetes and schizophrenia, compared to people with diabetes, but without schizophrenia (relative risk [RR] = 0.91, 95% confidence interval [CI] = 0.88–0.95) [10]. For example, people with schizophrenia were less likely to receive foot and eye examinations (RR = 0.96, CI = 0.93–0.99; RR = 0.97, CI = 0.94–0.99), blood pressure monitoring (RR = 0.98, CI = 0.96–0.99), and treatment with antihypertensive drugs (RR = 0.83, CI = 0.70–0.97) [10]. If a relatively large number of people with psychiatric disorders also have type 2 diabetes, this may put already vulnerable populations at increased risk for future health problems such as macro- and microvascular diseases.

Several systematic reviews have explored the prevalence of type 2 diabetes in people with different psychiatric disorders such as schizophrenia, major depression, bipolar disorder, and post-traumatic stress disorder (PTSD), indicating that the prevalence of type 2 diabetes is higher in people with psychiatric disorders, compared to people without psychiatric disorder [11–14]. To date, in a meta-analysis of 25 studies, Stubb et al. found that 9.5% of people with schizophrenia had type 2 diabetes and pooled relative risk compared to healthy control was 1.82 (95% CI 1.56–2.13, *n* = 4,489,125) [11]. Similarly, Vancampfort et al. estimated the prevalence of type 2 diabetes in people with major depression disorder, bipolar disorder, or PTSD to be 8.7–10% [12–14].

However, a comprehensive and systematic umbrella review with a critical appraisal of the existing literature into the prevalence of type 2 diabetes in people with different psychiatric disorders is lacking. Such an umbrella review of existing systematic reviews will not only help clarifying whether there are differences in the prevalence of type 2 diabetes in people with different psychiatric disorders, but will also contribute to evaluating the quality of the existing evidence.

### Objective

The umbrella review that we have planned to conduct aims to systematically summarize existing literature systematic reviews describing the prevalence of type 2 diabetes in people with a psychiatric disorder such as major depression, schizophrenia, PTSD, bipolar disorder, sleep disorder, or an anxiety disorder. Furthermore, when information is available in the identified systematic reviews, comparisons with control groups (from the general population or control groups without a psychiatric disorder) will be made. Finally, an assessment of the quality of the included systematic reviews will be conducted and discussed, providing directions for future research.

## Methods

This protocol is developed in accordance with PRISMA-P [Preferred Reporting Items for Systematic Reviews and Meta-Analyses Protocols] guidelines [15] (see Additional file 1). In accordance with the guidelines, the umbrella review is registered in PROSPERO (International Prospective Register of Systematic Reviews; registration number: ) in which any important protocol amendments will be noted.

### Search strategy

We plan to search in four electronic databases: Embase (1974 to present), PsycINFO (1967 to present), PubMed (1966 to present), and Cochrane Database of Systematic Reviews (1992 to present). Furthermore, reference lists from included papers will be manually screened for further eligible papers.

Four domains will be used in the following search: (I) psychiatric disorder, (II) prevalence, (III) diabetes, and (IV) systematic review. The search terms that capture the different psychiatric disorders will be taken from the DSM-III, DSM-IV-TR (axis I and axis II) [16, 17], DSM5 (section II) [18], and the ICD-10 Classification of mental and behavioral disorders (F00-F99) [19]. The four domains will be included in a four-step search combining relevant search words and MeSH terms with Boolean Logic operators (OR and AND). Search terms within each domain are combined with the operator “OR” and the different domains are combined with the operator “AND.” The search strategy has initially been targeted PubMed and will subsequently be adapted for the other databases. Additional file 2 shows the complete search string that will be used.

### Eligibility criteria

In the umbrella review, we will include systematic reviews focusing on cross-sectional and longitudinal observational studies in humans. The following eligibility criteria will be used:

i. A systematic review (with or without a meta-analysis), describing a systematic search string and eligibility criteria. Posters and abstracts describing a systematic review are not included.
ii. Focus on an adult population (≥ 18 years) with one or more psychiatric disorders (measured by, e.g., diagnostic interviews, hospital records, medical prescription, or self-reported measures). Psychiatric disorders include psychiatric diagnoses mentioned in the ICD or DSM classifications or elevated levels of clusters of psychiatric symptoms (DSM-III or DSM-IV, axis I or axis II; DSM-5 section II; ICD-10, F00–F99). Studies focusing on psychotropic medication that describe the prevalence of type 2 diabetes are also included. Studies that focus on a single psychiatric symptom and/or distress not described in the ICD or DSM classifications will not be included in the umbrella review (e.g., work-related stress and short sleep duration).
iii. Measure of prevalence of type 2 diabetes (measured by, e.g., diagnosis, medical reports, medical prescription, or self-reported measurement). If no information is available regarding subtypes of diabetes, we expect that the majority will have type 2 diabetes and include these studies.

Only publications in English, a Scandinavian language, German, or Dutch will be included. No exclusion criteria related to publication date will be set.

After removing duplicates, two independent reviewers will screen titles and abstracts to identity potential eligible papers. If at least one of the reviewers qualifies the systematic review as potentially eligible after reading the abstract, the full text will be retrieved and assessed according to the eligibility criteria. If there are any disagreements after the full-text screening, the two reviewers will discuss until consensus is reached. If necessary, a third reviewer will be involved in the discussions. Reference lists of the included publications will be manually screened with the aim to identify additional eligible publications. A PRISMA flowchart will describe the selection process. The screening of studies will be handled in EndNote and Covidence [20].

### Data extraction and data-synthesis

The data from the included systematic reviews will be extracted using a data extraction form that has been developed a priori. From each of the eligible systematic reviews the following information will be extracted: first author, year of publication, country, type of study, type of assessment of psychiatric disorder(s), type of assessment of type 2 diabetes, the number of studies included in the systematic review, total number of participants, and primary findings, including information on the prevalence of type 2 diabetes, odds ratios, and relative risk ratios, when possible. Furthermore, the number of cases and controls in each of the primary studies included in systematic reviews will be extracted. If the number of participants in the primary studies is not reported in the systematic reviews, the information will be retrieved from the papers describing these primary studies.

All systematic reviews will be summarized, and a narrative data-synthesis will be performed and presented for each of the psychiatric disorders identified in the umbrella review. Additionally, to summarize the reported prevalence of type 2 diabetes, we plan to conduct a meta-analysis of prevalence (a random effects model and 95% confidence interval) for each of the psychiatric disorders [21]. Our meta-analyses will be based on information from the primary studies included in the systematic reviews to make sure that the primary studies will only be included once [22]. We plan to assess between-study heterogeneity (quantified as *I*^2^ metric) [23, 24] and publication bias (small study effects, Egger’s test) [25].

### Risk of bias assessment

The strength of an umbrella review depends on the quality of the included systematic reviews. Therefore, it is important to assess the risk of bias in the included systematic reviews. Risk of bias assessment will be performed with the Risk of Bias in Systematic Reviews (ROBIS) tool [26].

## Discussion

The evidence from systematic reviews regarding the prevalence of type 2 diabetes in people with psychiatric disorders will be summarized. Furthermore, potential prevalence differences between people with psychiatric disorders and control groups without psychiatric disorders will be discussed and the implications for clinical care will be presented. When relevant, differences in assessment methodology will be discussed. If meaningful, the potential mechanisms explaining the prevalence of type 2 diabetes among individuals with a psychiatric disorder will be hypothesized and discussed. The quality of the included systematic reviews will be discussed, and implications presented. Finally, the results will most likely also be used to provide recommendations for future research.

## Supplementary information

**Additional file 1.** PRISMA-P checklist.

**Additional file 2.** Search strategy.

## Data Availability

Not applicable.

## Abbreviations

PRISMA-P: Preferred Reporting Items for Systematic Reviews and Meta-Analyses Protocols
RR: Relative risk
