# Supplementary material for: The prevalence of type 2 diabetes in people with psychiatric disorders: an umbrella review protocol
Source: Syst Rev. 2020 May 4;9:101. doi: 10.1186/s13643-020-01341-6 (PMC7199336; doi:10.1186/s13643-020-01341-6)
Supplement: Supplementary file 2 — Additional file 2. Search strategy. [file 13643_2020_1341_MOESM2_ESM.docx]

**Additional file 2. Search strategy**

| **PubMed** | | |
| --- | --- | --- |
| No. | Description or domain: | Search term |
| 1 | Search terms for psychiatric disorders (title/abstract) | psychiatric OR psychological* OR mental OR disorder* OR dysfunction* OR “developmental delay” OR psychogenetic OR stuttering OR autis* OR Asperger OR ADHD OR ADD OR pica OR encopresis OR enuresis OR “selective mutism” OR “acquired aphasia with epilepsy” OR “Rett´s syndrome” OR “disturbance of activity and attention” OR “elective mutism” OR cluttering OR delirium OR dementia OR Alzheimer OR amnesia OR “amnesic syndrome” OR hallucinosis OR “postencephalitic syndrome” OR “postconcussional syndrome” OR dependence OR abuse OR intoxication OR withdrawal OR “harmful use” OR schizophrenia OR psychosis OR psychoses OR psychotic OR catatonia OR depress* OR manic OR mania OR hypomania OR bipolar OR cyclothymi* OR dysthymi* OR agoraphobia OR phobi* OR “panic attack*” OR PTSD OR anxiety OR “predominantly obsessional thoughts or ruminations” OR OCD OR obsessions OR compulsions OR “predominantly compulsive acts” OR “acute stress reaction” OR “reaction* to severe stress” OR dissociate* OR hypochondriasis OR neurasthenia OR depersonalization OR “premature ejaculation” OR dyspareunia OR vaginismus OR paraphilia OR exhibitionism OR fetishism OR pedophilia OR “sexual masochism” OR “sexual sadism” OR voyeurism OR “sexual desire” OR “sexual aversion” OR “failure of genital response” OR “excessive sexual drive” OR transsexualism OR transvestism OR paedophilia OR sadomasochism OR “egodystonic sexual orientation” OR “inhibited female orgasm” OR “inhibited male orgasm” OR frotteurism OR anorexia OR bulimia OR overeating OR dyssomnia OR insomnia OR hypersomnia OR narcolepsy OR parasomnia OR hypoventilation OR “sleep apnea” OR “sleep walking” OR “sleep terror” OR “restless legs syndrome” OR “abuse of non-dependence-producing substances” OR “behavioural syndrome” OR dysphoria OR kleptomania OR pyromania OR “pathological gambling” OR trichotillomania OR excoriation OR retardation OR personality |
| 2 | MeSH terms for psychiatric disorders | "Mental Disorders"[Mesh] |
| 3 | Search terms for prevalence (title/abstract) | prevalence OR incidence OR epidemiology OR epidemiological OR “risk factor*” OR cross-sectional OR “cross sectional” |
| 4 | MeSH terms for prevalence | "Prevalence"[Mesh] OR "Epidemiology"[Mesh] OR "Incidence"[Mesh] OR "Epidemiologic Studies"[Mesh] |
| 5 | Search terms for diabetes (title/abstract) | diabetes |
| 6 | MeSH terms for diabetes | "Diabetes Mellitus"[Mesh] |
| 7 | Search terms for systematic review (title/abstract) | “systematic review*” OR “comprehensive review*” OR “systematic overview*” OR “comprehensive overview*” OR meta-analys* OR metaanalys* |
| 8 | MeSH terms for systematic review | "Review Literature as Topic"[Mesh] OR "Meta-Analysis as Topic"[Mesh] |
| 9 | Domain for psychiatric disorders | 1 OR 2 |
| 10 | Domain for prevalence | 3 OR 4 |
| 11 | Domain for diabetes | 5 OR 6 |
| 12 | Domain for systematic reviews | 7 OR 8 |
| 13 | Total search string | 9 AND 10 AND 11 AND 12 |

| **The Cochrane Database of Systematic Reviews** | | |
| --- | --- | --- |
| No. | Description or domain: | Search term |
| 1 | Search terms for psychiatric disorders (title/abstract) | psychiatric OR psychological* OR mental OR disorder* OR dysfunction* OR “developmental delay” OR psychogenetic OR stuttering OR autis* OR Asperger OR ADHD OR ADD OR pica OR encopresis OR enuresis OR “selective mutism” OR “acquired aphasia with epilepsy” OR “Rett´s syndrome” OR “disturbance of activity and attention” OR “elective mutism” OR cluttering OR delirium OR dementia OR Alzheimer OR amnesia OR “amnesic syndrome” OR hallucinosis OR “postencephalitic syndrome” OR “postconcussional syndrome” OR dependence OR abuse OR intoxication OR withdrawal OR “harmful use” OR schizophrenia OR psychosis OR psychoses OR psychotic OR catatonia OR depress* OR manic OR mania OR hypomania OR bipolar OR cyclothymi* OR dysthymi* OR agoraphobia OR phobi* OR “panic attack*” OR PTSD OR anxiety OR “predominantly obsessional thoughts or ruminations” OR OCD OR obsessions OR compulsions OR “predominantly compulsive acts” OR “acute stress reaction” OR “reaction* to severe stress” OR dissociate* OR hypochondriasis OR neurasthenia OR depersonalization OR “premature ejaculation” OR dyspareunia OR vaginismus OR paraphilia OR exhibitionism OR fetishism OR pedophilia OR “sexual masochism” OR “sexual sadism” OR voyeurism OR “sexual desire” OR “sexual aversion” OR “failure of genital response” OR “excessive sexual drive” OR transsexualism OR transvestism OR paedophilia OR sadomasochism OR “egodystonic sexual orientation” OR “inhibited female orgasm” OR “inhibited male orgasm” OR frotteurism OR anorexia OR bulimia OR overeating OR dyssomnia OR insomnia OR hypersomnia OR narcolepsy OR parasomnia OR hypoventilation OR “sleep apnea” OR “sleep walking” OR “sleep terror” OR “restless legs syndrome” OR “abuse of non-dependence-producing substances” OR “behavioural syndrome” OR dysphoria OR kleptomania OR pyromania OR “pathological gambling” OR trichotillomania OR excoriation OR retardation OR personality |
| 2 | MeSH terms for psychiatric disorders | MeSH descriptor: [Mental Disorders] explode all trees |
| 3 | Search terms for prevalence (title/abstract) | prevalence OR incidence OR epidemiology OR epidemiological OR risk factor* OR cross-sectional OR cross sectional |
| 4 | MeSH terms for prevalence | MeSH descriptor: [Prevalence] explode all trees OR MeSH descriptor: [Cross-sectional Studies] explode all trees |
| 5 | Search terms for diabetes (title/abstract) | diabetes |
| 6 | MeSH terms for diabetes | MeSH descriptor: [Diabetes Mellitus] explode all trees |
| 7 | Search terms for systematic review (title/abstract) | systematic review* OR comprehensive review* OR systematic overview* OR comprehensive overview* OR meta-analys* OR metaanalys* |
| 8 | MeSH terms for systematic review | MeSH descriptor: [Review Literature as Topic] explode all trees OR MeSH descriptor: [Meta-Analysis as Topic] explode all trees |
| 9 | Domain for psychiatric disorders | 1 OR 2 |
| 10 | Domain for prevalence | 3 OR 4 |
| 11 | Domain for diabetes | 5 OR 6 |
| 12 | Domain for systematic reviews/meta-analysis | 7 OR 8 |
| 13 | Total search string | 9 AND 10 AND 11 AND 12 |

| **PsycINFO** | | |
| --- | --- | --- |
| No. | Description or domain: | Search term |
| 1 | Search terms for psychiatric disorders  mp. (mp=title, abstract, heading eord, table of contents,key consepts, original title, tests & measures, mesh) | psychiatric OR psychological* OR mental OR disorder* OR dysfunction* OR developmental delay OR psychogenetic OR stuttering OR autis* OR Asperger OR ADHD OR pica OR encopresis OR enuresis OR selective mutism OR acquired aphasia with epilepsy OR Retts syndrome OR disturbance of activity and attention OR elective mutism OR cluttering OR delirium OR dementia OR Alzheimer OR amnesia OR amnesic syndrome OR hallucinosis OR postencephalitic syndrome OR postconcussional syndrome OR dependence OR abuse OR intoxication OR withdrawal OR schizophrenia OR psychosis OR psychoses OR psychotic OR catatonia OR depress* OR manic OR mania OR hypomania OR bipolar OR cyclothymi* OR dysthymi* OR agoraphobia OR phobi* OR panic attack* OR PTSD OR anxiety OR predominantly obsessional thoughts or ruminations OR OCD OR obsessions OR compulsions OR predominantly compulsive acts OR acute stress reaction OR reaction* to severe stress OR dissociate* OR hypochondriasis OR neurasthenia OR depersonalization OR premature ejaculation OR dyspareunia OR vaginismus OR paraphilia OR exhibitionism OR fetishism OR pedophilia OR sexual masochism OR sexual sadism OR voyeurism OR sexual desire OR sexual aversion OR failure of genital response OR excessive sexual drive OR transsexualism OR transvestism OR paedophilia OR sadomasochism OR egodystonic sexual orientation OR inhibited female orgasm OR inhibited male orgasm OR frotteurism OR anorexia OR bulimia OR overeating OR dyssomnia OR insomnia OR hypersomnia OR narcolepsy OR parasomnia OR hypoventilation OR sleep apnea OR sleep walking OR sleep terror OR restless legs syndrome OR abuse of non-dependence-producing substances OR behavioural syndrome OR dysphoria OR kleptomania OR pyromania OR pathological gambling OR trichotillomania OR excoriation OR retardation OR personality |
| 2 | Subject headings for psychiatric disorders | exp Mental Disorders/ |
| 3 | Search terms for prevalence  mp. (mp=title, abstract, heading eord, table of contents,key consepts, original title, tests & measures, mesh) | prevalence OR incidence OR epidemiology OR epidemiological OR risk factor* OR cross-sectional OR cross sectional |
| 4 | Subject headings for prevalence | exp Epidemiology/ OR exp Risk factor/ |
| 5 | Search terms for diabetes mp. (mp=title, abstract, heading eord, table of contents,key consepts, original title, tests & measures, mesh) | diabetes |
| 6 | Subject headings for diabetes | exp diabetes mellitus/ |
| 7 | Search terms for systematic review mp. (mp=title, abstract, heading eord, table of contents,key consepts, original title, tests & measures, mesh) | systematic review* OR comprehensive review* OR systematic overview* OR comprehensive overview* OR meta-analys* OR metaanalys* |
| 8 | Subject headings for systematic review | exp "literature review"/ OR exp meta analysis/ |
| 9 | Domain for psychiatric disorders | 1 OR 2 |
| 10 | Domain for prevalence | 3 OR 4 |
| 11 | Domain for diabetes | 5 OR 6 |
| 12 | Domain for systematic reviews/meta-analysis | 7 OR 8 |
| 13 | Total search string | 9 AND 10 AND 11 AND 12 |

| **Embase** | | |
| --- | --- | --- |
| No. | Description or domain: | Search term |
| 1 | Search terms for psychiatric disorders mp.  (mp. = title, abstract, heading word, drug trade name, original title, device manufacturer, drug manufacturer, device trade name, keyword, floating subheading word, candidate term word) | psychiatric OR psychological* OR mental OR disorder* OR dysfunction* OR developmental delay OR psychogenetic OR stuttering OR autis* OR Asperger OR ADHD OR pica OR encopresis OR enuresis OR selective mutism OR acquired aphasia with epilepsy OR Retts syndrome OR disturbance of activity and attention OR elective mutism OR cluttering OR delirium OR dementia OR Alzheimer OR amnesia OR amnesic syndrome OR hallucinosis OR postencephalitic syndrome OR postconcussional syndrome OR dependence OR abuse OR intoxication OR withdrawal OR schizophrenia OR psychosis OR psychoses OR psychotic OR catatonia OR depress* OR manic OR mania OR hypomania OR bipolar OR cyclothymi* OR dysthymi* OR agoraphobia OR phobi* OR panic attack* OR PTSD OR anxiety OR predominantly obsessional thoughts or ruminations OR OCD OR obsessions OR compulsions OR predominantly compulsive acts OR acute stress reaction OR reaction* to severe stress OR dissociate* OR hypochondriasis OR neurasthenia OR depersonalization OR premature ejaculation OR dyspareunia OR vaginismus OR paraphilia OR exhibitionism OR fetishism OR pedophilia OR sexual masochism OR sexual sadism OR voyeurism OR sexual desire OR sexual aversion OR failure of genital response OR excessive sexual drive OR transsexualism OR transvestism OR paedophilia OR sadomasochism OR egodystonic sexual orientation OR inhibited female orgasm OR inhibited male orgasm OR frotteurism OR anorexia OR bulimia OR overeating OR dyssomnia OR insomnia OR hypersomnia OR narcolepsy OR parasomnia OR hypoventilation OR sleep apnea OR sleep walking OR sleep terror OR restless legs syndrome OR abuse of non-dependence-producing substances OR behavioural syndrome OR dysphoria OR kleptomania OR pyromania OR pathological gambling OR trichotillomania OR excoriation OR retardation OR personality |
| 2 | Subject headings for psychiatric disorders | exp mental disease/ |
| 3 | Search terms for prevalence mp.  (mp. = title, abstract, heading word, drug trade name, original title, device manufacturer, drug manufacturer, device trade name, keyword, floating subheading word, candidate term word) | prevalence OR incidence OR epidemiology OR epidemiological OR risk factor* OR cross-sectional OR cross sectional |
| 4 | Subject headings for prevalence | exp Prevalence/ OR exp Epidemiology/ OR exp Incidence/ OR cross-sectional studies/ |
| 5 | Search terms for diabetes mp. (mp. = title, abstract, heading word, drug trade name, original title, device manufacturer, drug manufacturer, device trade name, keyword, floating subheading word, candidate term word) | diabetes |
| 6 | Subject headings for diabetes | exp diabetes mellitus/ |
| 7 | Search terms for systematic review mp. (mp. = title, abstract, heading word, drug trade name, original title, device manufacturer, drug manufacturer, device trade name, keyword, floating subheading word, candidate term word) | systematic review* OR comprehensive review* OR systematic overview* OR comprehensive overview* OR meta-analys* OR metaanalys* |
| 8 | Subject headings for systematic review | exp "systematic review"/ OR exp meta analysis/ |
| 9 | Domain for psychiatric disorders | 1 OR 2 |
| 10 | Domain for prevalence | 3 OR 4 |
| 11 | Domain for diabetes | 5 OR 6 |
| 12 | Domain for systematic reviews/meta-analysis | 7 OR 8 |
| 13 | Total search string | 9 AND 10 AND 11 AND 12 |
